# Setup uncertainties for inter-fractional head and neck cancer in radiotherapy

**DOI:** 10.18632/oncotarget.9748

**Published:** 2016-05-31

**Authors:** Eun-Tae Park, Sung Kwang Park

**Affiliations:** ^1^ Department of Radiation Oncology, Busan Paik Hospital, Inje University School of Medicine, Busan, Korea

**Keywords:** head and neck cancer, setup error, cervical spine, organ motion

## Abstract

**Purpose:**

The aim of this study is to determine the inter-fractional motion of cervical spine in radiotherapy (RT).

**Materials and Methods:**

Eleven localized head and neck cancer patients who were treated from April 2014 to September 2015 were evaluated. Every patient underwent 3 times of computed tomography (CT) simulation with equivalent setting. Left-right (LR, x) and antero-posterior (AP, z) directional shift of cervical spine were evaluated using 33 number of CT image. In regard to random error, geometric changes were evaluated by 22 data set (compared the first obtained CT to second or third CT) by one-sample *T* test. Systemic error was evaluated by each patients’ data set (11 pairs) by paired *T* test.

**Results:**

The mean random error of LR and AP translational shift of cervical spine were −0.39 ± 3.24 mm and −0.57 ± 0.99 mm respectively. The mean random error of translational change of AP direction showed statistical significance (*p* = 0.014). The mean random error of x and z rotational shift were −0.07 ± 0.29° and −0.05 ± 0.35°, respectively. The mean systemic error of translational shift of LR and AP direction were −0.64 ± 2.57 mm and −0.33 ± 1.22 mm, respectively. The mean systemic error of rotational shift of x and z were 0.01 ± 0.18° and −0.27 ± 0.33°, respectively. The mean systemic error of rotational changes of z direction showed statistical difference (*p* = 0.022).

**Conclusions:**

We have to be aware of the inter-fractional motion of cervical spine in head and neck RT and give enough margins in RT planning.

## INTRODUCTION

Currently, head and neck cancer radiotherapy (RT) is widely performed by intensity modulated radiotherapy (IMRT), which can produce steep dose gradient. Using IMRT, effective dose sparing to critical normal organ, such as spinal cord is capable. In practice, the setup corrections are mainly based on bony landmark, mostly cervical spine [1]. Although patients immobilized by thermoplastic head and neck mask, standard clear plastic headrests, and/or shoulder retractor [2], the location of cervical spine can be changed during the period of RT [3, 4]. Head and neck RT of curative intent generally take 6–7 weeks. Therefore, inter-fractional setup error can be commonly occurred in this long period of treatment time. Zhang et al. [5] previously reported about the setup uncertainties that the relative motion between C2 and C6 was in the range of 0.4 cm in one direction.

Setup error could bring on dose insufficiency of treatment target, whereas organ at risk such as spinal cord could receive excessive radiation dose [1]. Head and neck cancer are especially vunerable to dose insufficiency correlated locational changes due to its structural complexity [1, 6]. It could result in the poor treatment outcome, such as loco-regional failure and radiation induced toxicity such as myelopathy. Lower cervical spine (i.e., C5/C6/C7) which commonly included in RT of tumors originated from larynx and pharynx or metastatic cervical lymph nodes seems to be more movable than upper cervical spine [7].

Planning target volume (PTV) defined based on the geometric variation and setup inaccuracies of clinical target volume (CTV), which is the volume including gross tumor and subclinical microscopic disease. A formula was previously suggested by van Herk et al. [8]. This simple algorithm is generally accepted for choosing margins for PTV as follows:

To cover CTV for 90% of patients with 95% isodose:

PTV margin = 2.5·Σ + 0.7·σ

(Σ: Standard deviation of systematic error, σ: standard deviation of random error).

For given condition with a standard deviation of penumbra width as 3.2 mm, this recipe can be simplified to 2.5 times the standard deviation (SD) of preparation (systematic) errors plus 0.7 times the SD of execution (random) errors combined with the penumbra width.

Planning organ at risk volume (PRV) is a well known concept which produced from ICRU 62 report as volume given margins for organ at risk (OAR) [9]. Stroom et al. [10] previously reported the average margins recipe for spinal cord, based on the motion averaged maximum dose in the clinical target volume using van Herk's formula [8] as follows:

PRV margin (M_R_) = 1.6·Σ + 0.2·σ

The aim of this study is to find the inter-fractional setup uncertainties of cervical spine in head and neck radiotherapy. According this analysis, we try to suggest the proper margin of spinal cord in axial plane which is reflected daily setup error in radiation treatment planning.

## MATERIALS AND METHODS

### Patients and treatment

This study was conducted in 11 patients (9 men and 2 women) who underwent RT in Busan Paik Hospital from April 2014 to September 2015. Enrolled patients had non-metastatic head and neck cancer. Patient and treatment characteristics were summarized in Table 1. Nine patients (81.8%) were male and median age was 58 years (range 22–85). Primary tumors were located in nasopharynx (*n* = 4), larynx (*n* = 4), oropharynx (*n* = 2) and etc. Seven patients (63.6%) had regional cervical lymph node metastases at treatment. All metastatic lymph nodes were included in treatment target. Three patients (27.3%) underwent intensity-modulated RT. For radiotherapy, fraction size was median 2 Gy (range 2–2.5), and total dose was median 70 Gy (range 50–74).

**Table 1:** Patient and treatment characteristics (*n* = 11)

Characteristics	No. of patients	(%)
Age (yr)		
Median (range)	58 (22–85)	
Sex		
Male	9	(81.8)
Female	2	(18.2)
Primary site		
Nasopharynx	4	(36.4)
Larynx	4	(36.4)
Orophaynx	2	(18.2)
Unknown	1	(9.1)
Disease extent		
Locally invasive disease	4	(36.4)
Lymph node metastasis	7	(63.6)
Distant metastasis	0	(0)
Treatent modality		
3D CRT	8	(72.7)
IMRT	3	(27.3)
RT dose (Gy)		
Median (range)	70 (50–74)	

Abbreviations: 3D CRT = 3-dimensional conformal radiotherapy, IMRT = intensity modulated radiotherapy.

### Evaluation

All patients enrolled this study underwent 3 times of computed tomography (CT) simulation with equal setup, using the same immobilization device. In second and third CT simulations, equality of setup of left-right (LR) and antero-posterior (AP) direction was checked beforehand by laser beam and marking on the thermoplastic mask. The variation of the cervical spine location was evaluated by comparison from initial standard CT image to latter ones (second or third CT image). Therefore, 2 set (1 pair) of data of geometric changes were obtained from each patients. All 22 set (11 pairs) of data acquisited from 11 patients was used for evaluation.

**Figure 1:**
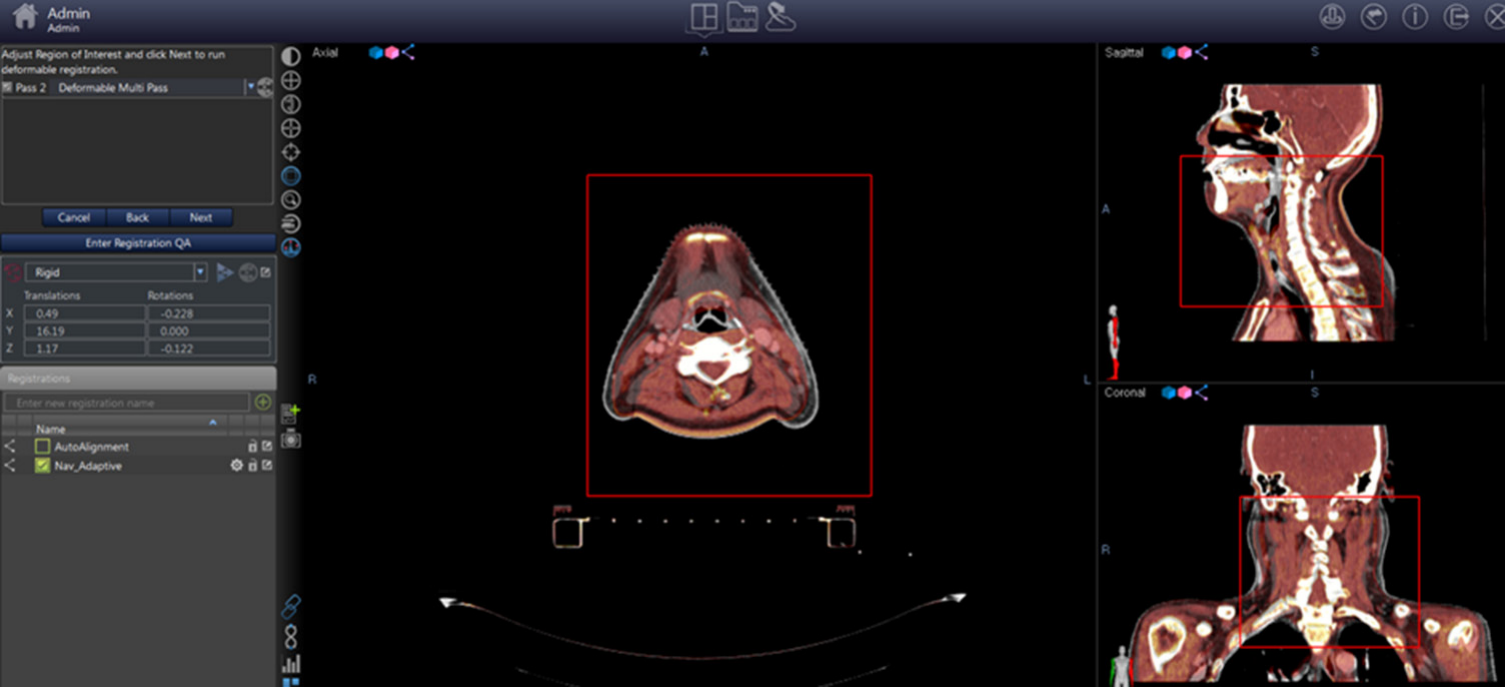
Registration setting in “adaptive monitoring”

VelocityAI 3.0 imaging software program provides the means to display, register and segment medical image volumes from multi modality source including CT, MRI, PET & SPECT images. VelocityAI 3.0 software was used for evaluation. In the function of segmentation, “adaptive monitoring” (one of the registration quality assurance programs) was used, which was a part of rigid registration. It was initially designed to compare the cone beam CT images to initial CT simulation image. This tool could be used to get information of the difference in location by image fusion guided by navigation. Information of locational changes was divided to translational error and rotational error (Figure 1).

In this study, we focused on evaluation to the LR (x) and AP (z) directional shift. Mean and standard deviation of setup error was checked. Superior-inferior (SI, y) setup equality was not checked before simulation CT and it could be largely influenced by CT image registration. Therefore, the superior-inferior directional shift (y) was ruled out on interpretation in discussion.

### Statistics

In regard to random error, inter-fractional changes were evaluated by 22 set of locational shift data (compared the first obtained CT to second or third CT) by one-sample *T* test. In regard to systematic error, each patients’ data set (11 pairs) was compared by paired *T* test. Spearman's rho correlation coefficient was used to find a correlation of serial locational changes of each patients. SPSS version 18 was used for statistical analysis. Statistical significance was defined as a *p*-value < 0.05.

## RESULTS

### Random error

Table 2 showed random error during RT. The random variation of LR (x), SI (y), and AP (z) translational shift of cervical spine were −0.39 ± 3.24 mm, 2.35 ± 14.30 mm, and −0.57 ± 0.99 mm respectively. The random variation of translational change of AP (z) direction showed statistical significance (*p* = 0.014). The random variation of rotational shift of x, y, and z axis were −0.07 ± 0.29°, −0.08 ± 0.45° and −0.05 ± 0.35°, respectively. There was no value of statistical significance in rotational shift.

**Table 2:** Random error(mm) by one sample *T* test (*n* = 22)

Variables	Mean	(range)	SD	*p*-value
Translation				
x	−0.39	(−6.23∼9.41)	3.24	0.578
y	2.35	(−28.68∼26.61)	14.30	0.450
z	−0.57	(−2.91∼1.20)	0.99	0.014
Rotation				
x	−0.07	(−0.69∼0.39)	0.29	0.286
y	−0.08	(−0.90∼1.13)	0.45	0.439
z	−0.05	(−0.81∼0.72)	0.35	0.500

Abbreviations: x = Left-right axis, y = Superior-inferior axis, z = Antero-posterior axis, and SD = standard deviation.

### Systemic error

Serial changes of location which was calculated by paired *T* test were summarized in Table 3. In comparison of locational changes of first-second CT images with first-third CT images, the systematic variation of translational shift of LR (x), SI (y), and AP (z) direction were −0.64 ± 2.57 mm, −0.78 ± 13.17 mm, and −0.33 ± 1.22 mm, respectively. For the systematic variation, rotational shift of x, y, and z were observed as 0.01 ± 0.18°, 0.39 ± 0.33°, and −0.27 ± 0.33°, respectively. The systematic variation of rotational changes of AP direction significantly different (*p* = 0.022).

**Table 3:** Systemic error(mm) in each patients’ data by paired *T* test (11 pairs)

Variables	Mean	SD	*p*-value	95% C.I.
Translation				
x	−0.64	2.57	0.431	(−2.36∼1.09)
y	−0.78	13.17	0.848	(−9.63∼8.07)
z	−0.33	1.23	0.392	(−1.15∼0.49)
Rotation				
x	0.10	0.19	0.861	(−0.12∼0.14)
y	0.39	0.33	0.003	(0.17−0.62)
z	−0.27	0.33	0.022	(−0.49∼−0.05)

Abbreviations: x = Left-right axis, y = Superior-inferior axis, z = Antero-posterior axis, and SD = standard variation.

### Correlation

Correlation of serial locational changes also evaluated by Spearman correlation was presented in Table 4. Changes in translational x and rotational x direction were significantly correlated between first-second and first-third CT images (*p* < 0.001 and *p* = 0.021). Although, translational changes of y direction was not correlated between two groups (*p* = 0.052), rotational changes of y direction showed significant correlation (*p* < 0.001). The changes in translational z and rotational z direction were not significantly correlated (*p* = 0.473 and *p* = 0.061).

**Table 4:** Correlation of geometric changes in each patients’ data (11 pairs)

Variables	Correlation coefficient	*p*-value
Translation		
x	0.927	< 0.001
y	0.598	0.052
z	0.242	0.473
Rotation		
x	0.680	0.021
y	0.890	< 0.001
z	0.591	0.061

### Subgroup analysis for random and systemic error according to cervical spine level

We classified cervical spine into 3 groups: C1-2, C3-5, and C6-7. Subgroup analysis was performed according to the level of cervical spine, respectively.

As for the random error (Table 5), translational shift of x direction showed significant changes in all level of cervical spine (C1-2 *p* = 0.004, C3-5 *p* = 0.024, and C6-7 *p* = 0.040). Translational shift of z direction showed significant changes in upper cervical spine (C1-2 *p* = 0.034). However, the changes of mid and lower cervical spine was not significant. Upper cervical spine (C1-2) showed significantly change in rotational shift of z. Lower cervical spine (C6-7) also showed significant change in rotational shift of x (*p* = 0.006).

**Table 5:** Subgroup analysis of random error(mm) according to cervical spine level

Variables	C1-2			C3-5			C6-7		
	mean	SD	*p*-value	mean	SD	*p*-value	mean	SD	*p*-value
Translation									
x	−2.01	2.96	0.004	−1.34	2.58	0.024	−1.33	2.86	0.040
y	1.45	14.6	0.646	1.38	14.71	0.665	0.39	14.42	0.902
z	−0.50	1.03	0.034	0.02	0.91	0.919	−0.18	0.81	0.314
Rotation									
x	0.31	0.917	0.130	−0.09	0.33	0.203	−0.29	0.45	0.006
y	−0.46	0.591	0.001	−0.35	0.43	0.001	−0.20	0.35	0.013
z	−0.89	1.160	0.002	0.01	0.30	0.893	0.12	0.56	0.333

In contrast, there was no statistical significant changes in systemic error according to level of cervical spine (Table 6).

**Table 6:** Subgroup analysis of systemic error(mm) according to cervical spine level

Variables	C1-2			C3-5			C6-7		
	mean	SD	*p*-value	mean	SD	*p*-value	mean	SD	*p*-value
Translation									
x	0.56	4.23	0.671	0.85	2.98	0.366	−0.07	2.35	0.922
y	2.88	11.8	0.436	1.9	11.60	0.598	−1.23	17.64	0.822
z	−0.18	1.09	0.595	−0.11	1.10	0.746	0	0.79	0.988
Rotation									
x	−0.44	0.82	0.108	−0.07	0.36	0.509	−0.10	0.40	0.450
y	0.27	0.75	0.262	0.37	0.60	0.064	−0.02	0.36	0.861
z	−0.04	0.88	0.869	−0.31	0.37	0.020	−0.14	0.49	0.367

## DISCUSSION

According to our study result, inter-fractional setup error prominently existed in head and neck RT. Basis of Stroom's formula [10] about PRV margin, proper margins for x and z direction were calculated to 5.6 mm and 1.8 mm, respectively. Although there's no standard concept of PRV margin [11] of head and neck cancer, general margin for spinal cord seems to be required to maintain the safety of RT. Motion of spine might be different to motion of target volume. Small axial displacement of spine could result in inappropriate dose distribution of target.

In the subgroup analysis of random error according to cervical spine level, upper cervical spine (C1-2) showed significant changes in both translational x and z direction, whereas lower cervical spine (C6-7) showed significant changes only in translational x direction (Table 5). We should give more attention to the unexpected movement of upper cervical spine during RT.

Contouring the interest region (i.e., metastatic cervical nodes) could bring more data about physical displacement or dose coverage [12]. Evaluation with dose volume histogram could give more meaningful clinical information [13–15]. Further exquisite evaluation should be performed in this regard.

As for limitations, patients’ number of current study is too small to make firm any conclusions. Especially, analysis of systemic error based on only 11 pairs of data sets. A small number of data has limitation on statistical analysis and further interpretation.

Directional change of y direction of this study was too various and we did not give meaning on that because checking the SI movement in CT was impossible in the setting of CT simulation. Although we could not evaluate y axis movement in this study, well-designed, prepared study protocol to check y axis changes could find the sagittal plane movement.

We did not deal with the intra-fractional changes of location in this study. Motion of body during RT also largely account for the setup error [16, 17]. Real time correction might be warranted to make a more precise treatment and further study for intra-fractional changes is required.

In conclusion, we have to be aware of the inter-fractional setup error in RT. LR (x) directional changes of cervical spine is larger than we recognized. Therefore, we should give enough margins to spinal cord in RT planning.
